# Intracellular Biomacromolecule Delivery by Stimuli-Responsive Protein Vesicles Loaded by Hydrophobic Ion Pairing

**DOI:** 10.1021/acsomega.4c07666

**Published:** 2025-01-14

**Authors:** Mikaela A. Gray, Alejandro de Janon, Michelle Seeler, William T. Heller, Nicki Panoskaltsis, Athanasios Mantalaris, Julie A. Champion

**Affiliations:** † Chemical and Biomolecular Engineering, Georgia Institute of Technology Atlanta, Georgia 30332-0002, United States; ‡ Biomedical Systems Engineering Laboratory, Georgia Institute of Technology, Atlanta, Georgia 30332-0002, United States; § School of Biological Sciences, Georgia Institute of Technology, Atlanta, Georgia 30332-0002, United States; ∥ Neutron Scattering Division, Oak Ridge National Laboratory, Oak Ridge, Tennessee 37831-6473, United States; ⊥ School of Pharmacy and Pharmaceutical Sciences, Trinity College Dublin, Dublin 2, Ireland; # Department of Haematology, St. James’s Hospital, Dublin 8, Ireland; ∇ National Institute for Bioprocessing Research and Training, Dublin A94 X099, Ireland

## Abstract

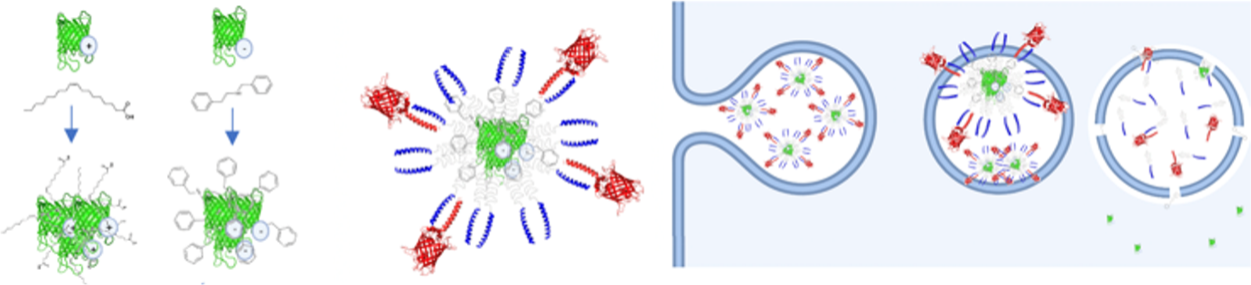

Proteins can perform ideal therapeutic functions. However, their large size and significant surface hydrophilicity and charge prohibit them from reaching intracellular targets. These chemical features also render them poorly encapsulated by nanoparticles used for intracellular delivery. In this work, a novel combination of protein vesicles and hydrophobic ion pairing (HIP) was used to load protein cargo and achieve cytosolic delivery to overcome the limitations of previous protein vesicle properties. Protein vesicles are thermally self-assembling nanoparticles made from elastin-like polypeptide (ELP) fused to an arginine-rich leucine zipper and a globular protein fused to a glutamate-rich leucine zipper. To impart stimuli-responsive disassembly, physiological stability, and small size, the ELP sequence was modified to include histidine and tyrosine residues. HIP was used to load and release protein cargo requiring endosomal escape for cytosolic function. HIP vesicles enabled delivery of cytochrome c, a cytosolically active protein, and a significant reduction in viability in both a traditional two-dimensional (2D) human cancer cell line culture and a biomimetic three-dimensional (3D) organoid model of acute myeloid leukemia. By examining the uptake of positively and negatively charged fluorescent protein cargos loaded by HIP, this work revealed the necessity of HIP for cytosolic cargo delivery and how HIP loading influences protein vesicle self-assembly and disassembly using microscopy, small-angle X-ray scattering, and nanoparticle tracking analysis. HIP protein vesicles have the potential to broaden the use of intracellular proteins as therapeutics for various diseases and extend protein vesicles to deliver other biomacromolecules, as the strategy developed here resulted in the first cytosolic protein cargo delivery using protein vesicles.

## 1 Introduction

Intracellular protein therapeutics could have high potency and specificity against diseases such as cancer by directly engaging in cell signaling pathways or metabolism.^1−3^ With these advantages, they have the potential to exhibit less toxicity to healthy tissue than small molecule drugs.^2,4,5^ However, there are significant delivery challenges for intracellular protein therapeutics: instability following administration due to degradation by proteases or unfolding, immunogenicity, and inability to cross cell membranes.^6,7^ Nanoparticles such as liposomes, polymeric nanoparticles, and protein nanoparticles encapsulate proteins to protect them from degradation, can be decorated to allow for targeted delivery, improve endocytic delivery, and provide controlled release.^8−10^ Liposome and polymeric nanoparticles often use organic solvents during fabrication, and some formulations possess toxicity-related concerns. Organic solvents can denature or change the native structure of a protein by disrupting hydrophobic interactions, which leads to loss of protein function.^11^ One common challenge many nanoparticles face is poor encapsulation of large, charged biomacromolecule cargos because most nanoparticles were developed for smaller and more hydrophobic cargos and their building blocks tend to be smaller than proteins. When encapsulating protein cargos, many nanocarriers have low encapsulation efficiency (EE), undesired cargo leakiness, and an initial cargo burst release.^12^

To overcome the challenges associated with encapsulating biomacromolecule cargos, hydrophobic ion pairing (HIP) has been used, which reduces the solubility of compounds by forming reversible ionic interactions between a charged therapeutic and an oppositely charged hydrophobic counterion.^13^ HIP decreases the electrostatic self-repulsion of the therapeutic by neutralizing the molecule’s natural surface charge and increases the hydrophobicity by coating the molecule’s surface with hydrophobic domains.^14^ Together these effects lead to the formation of complexes of multiple therapeutics and hydrophobic ions that can then be encapsulated by a hydrophobic nanoparticle component. HIP is reversed when the therapeutic and counterion are dissociated by competition from other ions due to a change in pH or salt concentration resulting in a slower rate of cargo release from nanoparticles.^15^ Nanoparticles are exposed to increasingly acidic environments from weakly acidic endosomes (pH 5.5–6.0) to more acidic lysosomes (pH 4.5–5.0) that cargo must usually escape to achieve cytosolic delivery.^16^ Ionizable lipids such as ALC-0315, used in the Pfizer/BioNTech SARS-CoV-2 mRNA lipid nanoparticle vaccine, are designed to capitalize on endosomal acidification to deliver mRNA into the cytosol.^17^ Sun and co-workers showed that pairing insulin and hydrophobic ion oleic acid in pH-sensitive nanoparticles increased the EE of insulin from 42.8 to 94.6%.^18^ HIP has been utilized to load and deliver small molecules, peptides, and proteins, including insulin, polymyxin B, and lysozyme, in various types of nanoparticles.^13^ HIP complexes improve membrane permeability because of their high solubility in the cell membrane and improve the oral bioavailability of many drugs.^19^

We propose that self-assembling protein vesicles, which are genetically tunable, biodegradable nanoparticles made without solvent, could be used for cytosolic biomacromolecule delivery.^20^ Protein vesicles consist of a globular protein (mCherry in this work) attached to a glutamic acid-rich leucine zipper (Z_E_) that binds strongly to an arginine-rich leucine zipper (Z_R_) attached to an elastin-like polypeptide (ELP) to form a protein complex. Above the transition temperature (*T*_t_), ELP undergoes a conformational change from soluble to insoluble depending on salt concentration and type, Z_E_/Z_R_ molar ratio (always <1), ELP sequence, and protein concentration.^21−26^ Our group has demonstrated doxorubicin HCl loading and release in vitro from covalently cross-linked protein vesicles.^27^ However, the cross-linking used to stabilize the vesicles in physiological conditions limits the size of cargo that can be released from protein vesicles. Recently, we rationally designed an ELP variant to produce stable nanoscale protein vesicles at physiological salt concentration without cross-linking.^21^ This was accomplished by increasing ELP hydrophobicity by substituting tyrosine residues (Y) for valine residues (V) at 5 guest positions (X) in the ELP sequence (VPGXG)_25_ (P = proline, G = glycine), which decreases the *T*_t_, thereby reducing the time in the growth phase and subsequent vesicle diameter and reducing the required salt for ELP transition and vesicle formation. Additionally, we inserted ionizable histidine residues (H) to impart pH-responsiveness to ELP, evidenced by vesicle disassembly in acidic conditions, to aid in escaping the endosome.^28^ We hypothesize that combining the pH-sensitive ELP and the hydrophobic ELP will form protein vesicles that both stably assemble in a physiological salt concentration and disassemble with an acidic pH trigger.

Motivated by HIP encapsulation of therapeutics into polymer and lipid nanoparticles and the ability of protein vesicles to encapsulate and deliver hydrophobic small molecules,^13,27,29^ this work examined how pH-sensitive protein vesicles can be combined with HIP for encapsulation, release, and cytosolic delivery of charged protein cargos. Two commonly used, generally regarded as safe (GRAS), anionic counterions were selected: oleic acid (OA) and sodium docusate (SD). OA is a fatty acid used in cholesterol supplements and is a known transdermal permeation enhancer.^30^ SD is a laxative that decreases surface tension.^31^ In an oral delivery study of leuprorelin, insulin, and desmopressin peptides using HIP with self-emulsifying drug-delivering systems, SD showed the best EE.^32^ Benethamine (BA) was selected as the cationic counterion. Most cations are toxic, but BA is a lipophilic amine used as a drug salt in FDA-approved penicillin G, a long-acting penicillin. BA has been paired with retinoic acid for improved EE in a nanostructured lipid carrier compared to two other cationic counterions.^33^ Here, we develop a formulation of protein vesicles using engineered proteins and HIP to intracellularly deliver functional protein cargo, expanding the cargo types used for protein vesicle delivery.

## 2 Results and Discussion

### 2.1 Protein Vesicles Made from Rationally Designed ELPs

**Figure 1:**
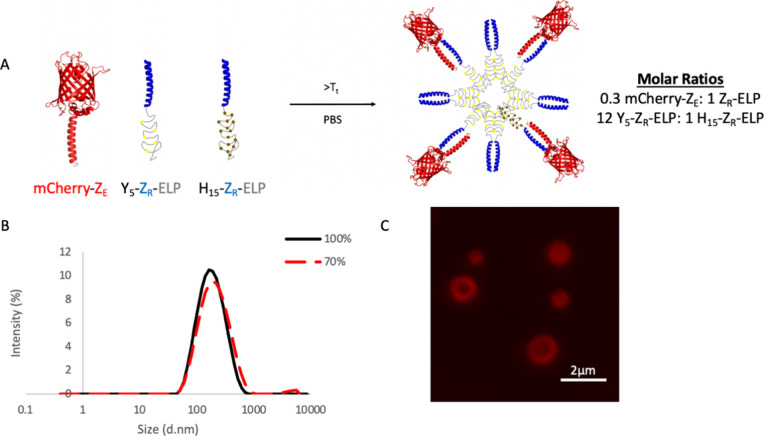
Protein vesicle characterization. (A) Illustration of self-assembling protein building blocks composed of mCherry-Z_E_ and a mixture of Z_R_-ELPs using a 0.3 mCherry-Z_E_/Z_R_-ELP ratio and 30 μM total ELP using a 12 Y_5_-Z_R_-ELP (labeled with yellow spheres):1 H_15_-Z_R_-ELP (labeled with brown spheres) molar ratio with 0.15 M NaCl. (B) DLS analysis showing the size distribution of vesicles and stability of vesicles when diluted to 70% of original concentration into PBS. (C) Epifluorescence image of structures composed of mCherry-Z_E_ and a mixture of Z_R_-ELPs using a 0.05 Z_E_/Z_R_ ratio for enhanced visualization and 30 μM total ELP using a 12 Y_5_-Z_R_-ELP: 1 H_15_-Z_R_-ELP molar ratio in 0.15 M NaCl. The image was digitally enlarged 10×.

For this work, a pH-sensitive ELP with 15 histidine residues (H_15_-Z_R_-ELP) and an ELP with 5 tyrosine residues (Y_5_-Z_R_-ELP) were selected to be expressed as fusion proteins with Z_R_ in *Escherichia coli* (*E. coli*) and purified (Table S1 and Figure S1). The ionization of histidine at acidic pH increases the *T*_t_, inducing the ELP to reverse the transition from hydrophobic back to soluble, which destabilizes the vesicles. Addition of tyrosine decreases the *T*_t_, thereby reducing the required salt for ELP transition and vesicle formation to a physiological concentration and reducing the time in the growth phase and subsequent vesicle diameter. To visualize vesicles, model fluorescent protein mCherry-Z_E_ was selected as the globular protein, expressed as a fusion to Z_E_ in *E. coli*, and purified. The proteins were mixed in phosphate-buffered saline (PBS) and warmed to 25 °C for 1 h to form vesicles (Figure 1A). A 12 Y_5_-Z_R_-ELP: 1 H_15_-Z_R_-ELP molar ratio was selected over 2:1, 1:1, and 1:0 ratios as it formed vesicles with the smallest size and a measurable amount of H_15_-Z_R_-ELP without aggregation using a 0.05 Z_E_/Z_R_ molar ratio (Figure S3). There was no size difference in doubling the Y_5_-Z_R_-ELP content from a ratio of 1:1 to 2:1, so a large increase to 12:1 was made to ensure a nanoscale size with low polydispersity. As in previous work,^21^ increasing the Z_E_/Z_R_ molar ratio to 0.3 further reduced the vesicle diameter to 141.2 ± 3.64 nm by dynamic light scattering (DLS) with a polydispersity index (PDI) of 0.291 ± 0.03 (Figure 1B). As the vesicles were too small to observe their morphology by fluorescence microscopy, we examined the turbidity profile (Figure S4). The turbidity remained high after the initial increase as is characteristic of stable vesicles; coacervates, which are an intermediate phase in the vesicle assembly process, display decreasing turbidity after the initial increase.^20,34^ Vesicle morphology was examined using a lower Z_E_/Z_R_ ratio (0.05), to make large structures visible using epifluorescence, where the particles were hollow and spherical (Figure 1C). The zeta potential of this formulation of protein vesicles was −9.76 ± 1.9 mV, approximately neutral to slightly negative. The vesicles maintained a stable diameter when diluted in PBS by 30% (the maximum for high enough concentration for DLS), showing that a reduction in protein concentration did not induce disassembly (Figure 1B).

### 2.2 Protein Vesicles Deliver Cytochrome c Loaded by HIP to the Cytosol

In order to determine if protein vesicles made from tyrosine and histidine ELPs can deliver a functional protein to the cytosol, cytochrome c (CytC) was selected as the cargo. CytC induces apoptosis when localized in the cytosol of the cell, so cargo trapped in vesicles or endosomes would not reduce cell viability or be interpreted as successful delivery.^35−37^ CytC has an isoelectric point of 12.4, so it is positively charged at physiological and acidic pH and was paired with OA (Figure S2A, p*K*_a_ 5.02) and SD (Figure S2B, p*K*_a_ −0.75) anions. The charge ratios were calculated by using the molar ratio of protein and counterion and a surface potential calculator to determine the total protein surface potential relative to the counterion, which has 1 anionic group.^38^ To determine if the amount of counterion influenced the size of protein vesicles, the concentration of CytC was kept constant, while the amount of OA counterion was varied (Figure S5A). The concentration of OA and protein vesicle size were linearly correlated, forming nano- to micrometer-sized particles as OA increased. The change in vesicle size could be due to hydrophobic interactions between the ELP and counterions, influencing coacervate size.

**Figure 2:**
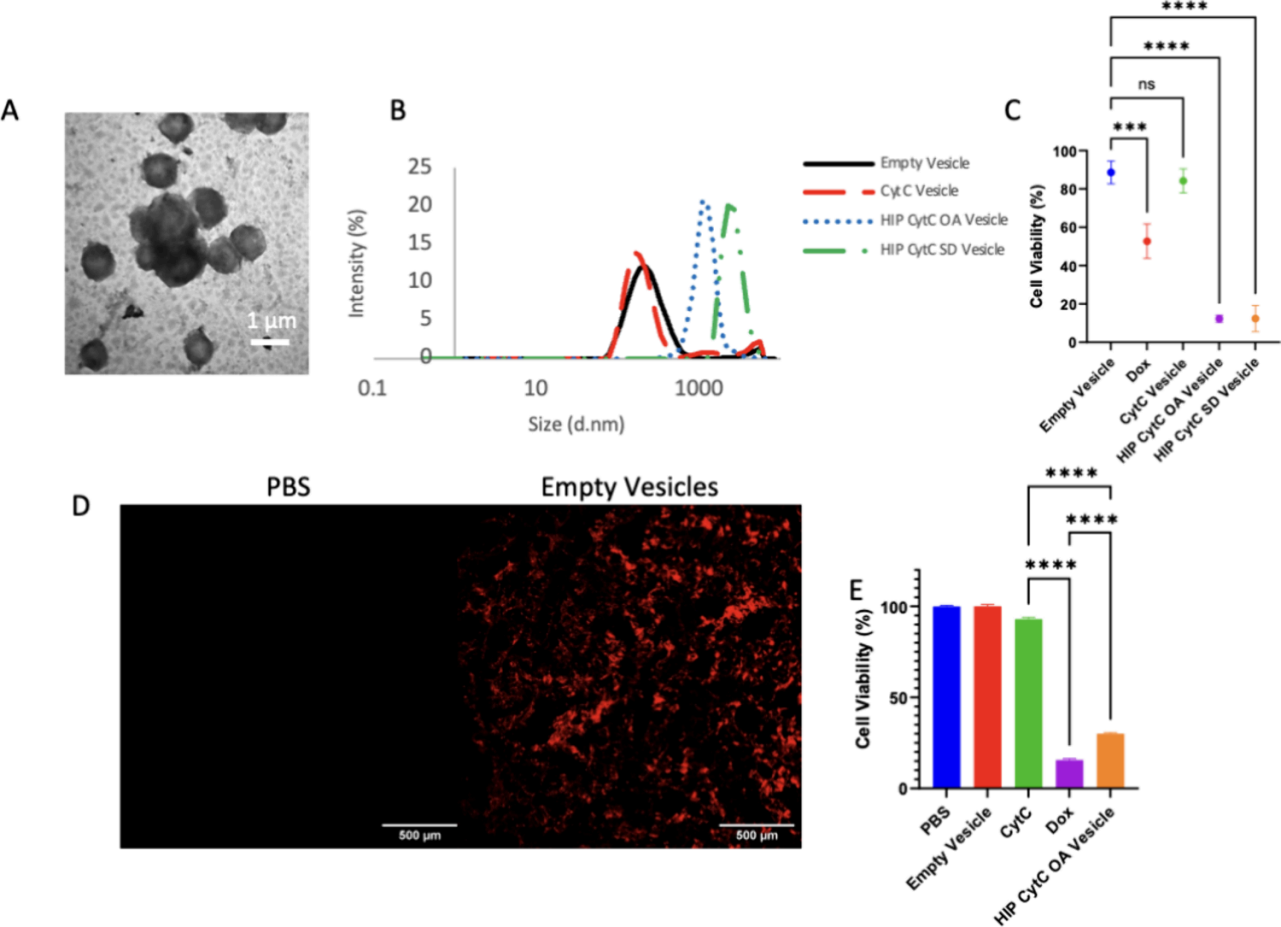
CytC vesicle characterization and delivery. (A) TEM of HIP CytC OA-loaded vesicles composed of mCherry-Z_E_ and a mixture of Z_R_-ELPs using a 0.3 Z_E_/Z_R_ ratio, 30 μM total ELP with a 12 Y_5_-Z_R_-ELP: 1 H_15_-Z_R_-ELP molar ratio, 10 μM CytC cargo, and 0.15 M NaCl. (B) DLS analysis showing the size distribution of cargo-loaded vesicles. (C) HeLa cell viability measured by an MTT assay 48 h after treatment with 2 μM CytC relative to media control. (D) Vesicle diffusion within an acute myeloid leukemia organoid. (E) Acute myeloid leukemia bone marrow organoid K562 cell viability 24 h after treatment with 2 μM CytC or 6 μM Dox showing the ability to enter the cytosol of cells in a tumor-like environment. One-way ANOVA was utilized with *p* > 0.05 n.s., *p* < 0.001***, *p* < 0.0001****, and *n* = 3 groups with each experiment repeated at least twice. Error bars are standard deviation from the mean.

Properties of HIP CytC OA- or SD-loaded vesicles are listed in Table 1 describing the EE, vesicle size, and PDI. The charge ratios of HIP OA or SD complexes listed in Table 1 resulted in no significant reduction in CytC fluorescence, demonstrating retention of the protein structure (Figure S6A). After using HIP to load CytC into protein vesicles, vesicles were incubated with cells for 48 h, and cell viability was measured as a loss in viability would indicate cytosolic delivery. First, we tested charge ratios to determine their effect on cytosolic delivery. A charge ratio of 1 CytC: 1.8 OA or 1 CytC: 5.33 OA did not result in cytosolic delivery (Figure S5B,C). However, a 1 CytC: 16 OA charge ratio yielded significant cytosolic delivery in K562 acute myeloid leukemia cells (Figure S5). Other researchers using HIP to load drugs into nanoparticles used significantly lower charge ratios, typically 1 cargo (small hydrophobic molecules, peptides, and proteins):1 counterion, as higher charge ratios formed micelles in their complexes.^32,39−42^ No micelle structures were observed with vesicles by DLS (Figures 2B and S7). We hypothesize that lower ratios are insufficient for altering the hydrophobicity of the CytC surface as OA molecules could insert their tails into the protein core to reduce water exposure, which would instead increase the total surface charge and polarity of CytC. Additionally, we posit that excess counterion aids in cargo endosomal escape as more OA will disassociate from the protein and interact with the endosomal membrane upon reduction in pH and changes in osmolarity.^43^ We also observed less than 5% viability for 1 CytC: 16 OA or 1 CytC: 13 SD HIP loaded protein vesicles with HeLa cells (Figure 2C). OA has a higher LogP value (6.78) indicating greater hydrophobicity than SD (5.2); however, both counterions enabled cytosolic delivery for charge ratios of 1 CytC:13 SD or 1 CytC:16 OA with these monovalent counterions. HIP loaded vesicles with nontoxic cargo (data in Section 2.3) do not reduce cell viability, confirming that the loss in viability is due to delivery of CytC, not toxicity from delivery of hydrophobic ions. Vesicles loaded with CytC without HIP do not influence viability at all despite having EEs similar to those of HIP loaded vesicles. While EE is a traditional parameter to improve with a new formulation, the improvement provided by HIP is functional cytosolic delivery; without HIP, CytC is not delivered to the cytosol. However, the high charge ratios required for successful delivery do increase the vesicle size. It was surprising that larger vesicles delivered CytC so well, when nanoscale particles typically deliver better.^8^ It may be related to the coacervate nature of ELP materials as Lim and co-workers demonstrated that micron-sized peptide coacervate droplets were effective for intracellular delivery applications, though the droplets cross the cell membrane in an energy-independent fashion, not via endocytosis like vesicles.^44^ We confirmed that increasing vesicle size was not responsible for CytC delivery, as neither nanoscale nor microscale vesicles loaded with CytC without HIP reduced viability (Figure S5D).

**Table 1:** Molar and Charge Ratios of Cargo to Hydrophobic Counterion, Vesicle Size, PDI, and EE Reported as Mean ± Standard Deviation^a^

cargo	counterion	molar ratio	charge ratio	vesicle size (nm)	PDI	EE (%)
CytC	none			203 ± 8	0.32 ± 0.03	46.85 ± 5.82
	OA	1:127	1:16	1155 ± 158***	0.12 ± 0.08*	41.21 ± 2.46
	SD	1:108	1:13	3485 ± 1130**	0.19 ± 0.07*	52.79 ± 3.90
sfGFP(+10)	none			261 ± 5	0.22 ± 0.01	64.09 ± 4.49
	OA	1:396	1:35	174 ± 1***	0.26 ± 0.01**	98.16 ± 1.43***
	SD	1:62	1:5.4	n/m	n/m	n/m
sfGFP(−10)	none			216 ± 17	0.29 ± 0.05	16.52 ± 1.65
	BA	1:132	1:14	193 ± 14	0.35 ± 0.05	20.37 ± 2.04

a n/m means values were not measurable. * indicates unpaired *t*-test comparison between no counterion and HIP groups (*p < 0.05, **p < 0.01, ***p < 0.001, or ****p < 0.0001).

To measure therapeutic delivery efficacy in a complex tumor-like environment, an acute myeloid leukemia organoid (using the K562 cell line) was utilized. Acute myeloid leukemia is a bone marrow cancer characterized by expansion and differentiation arrest of myeloid progenitor cells, with a 5-year survival of 24%.^45^ This biomimetic bone marrow organoid, containing collagen I, replicates the structural characteristics of human bone marrow and captures various biological cues unique to this tissue, exhibiting comparable porosity, pore size distribution, and stiffness, allowing cells to faithfully recreate spatial niches reminiscent of in vivo systems.^46^ Vesicles penetrated the 100–200 μm-sized niche pores within the organoid (Figure 2D).^46^ Vesicles delivered the cargo intracellularly resulting in less than 35% cell viability using a 2 μM dose of CytC after 24 h of treatment (Figure 2E). This is significant because organoids have self-organization of acute myeloid leukemia niches and mirror leukemic metabolism yielding an environment more like a human acute myeloid leukemia bone marrow tumor microenvironment compared to traditional 2D cell cultures.^46,47^

### 2.3 Characterization of Vesicles Loaded with Model Positive and Negative Protein Cargos

**Figure 3:**
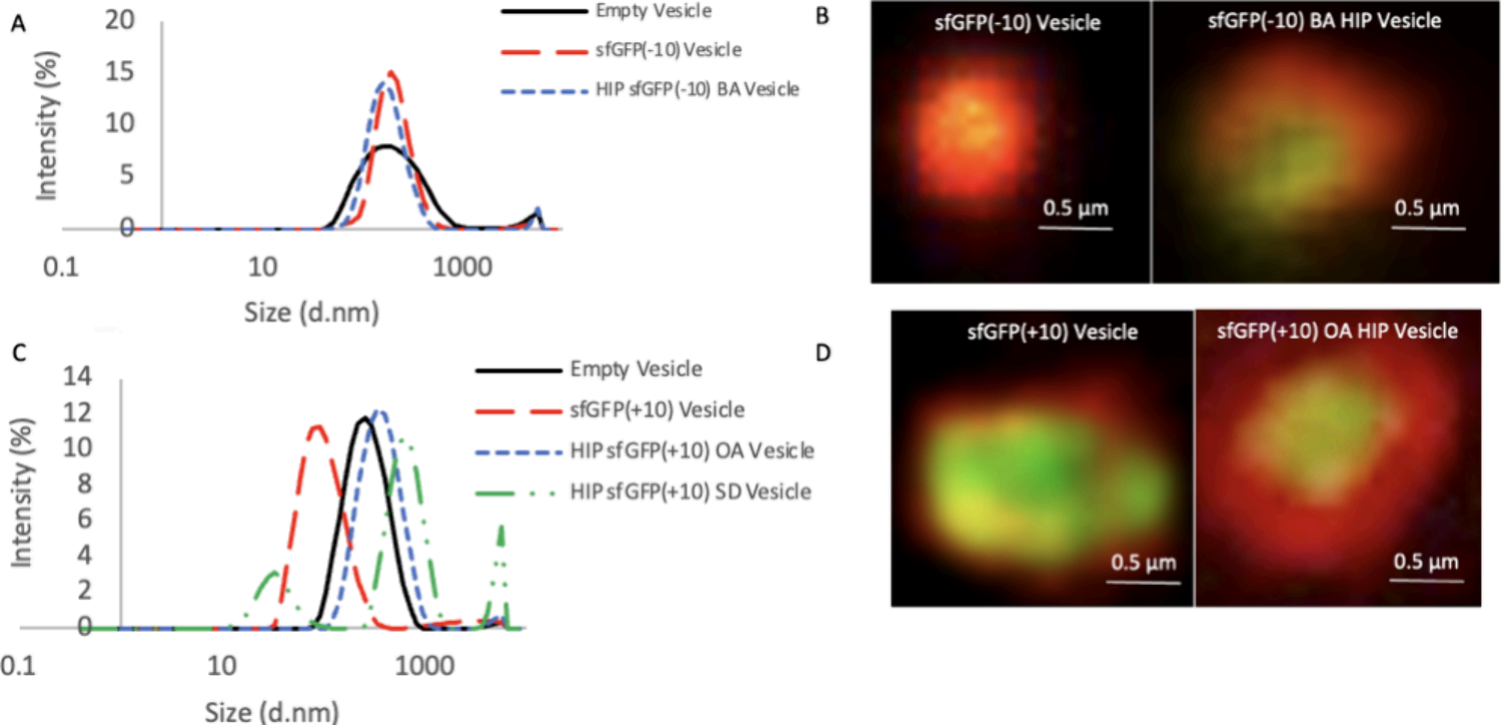
Empty, sfGFP(−10), and sfGFP(+10) loaded vesicle characterization. (A) DLS size distribution of cargo-loaded vesicles composed of mCherry-Z_E_ and a mixture of Z_R_-ELPs using a 0.3 Z_E_/Z_R_ ratio, 30 μM total ELP using a 12 Y_5_-Z_R_-ELP: 1 H_15_-Z_R_-ELP molar ratio with and without 10 μM of sfGFP(−10) cargo in 0.15 M NaCl or with HIP sfGFP(−10) BA. (B) Epifluorescence images of structures composed of larger vesicles using a 0.05 Z_E_/Z_R_ ratio (all other conditions are the same as (A) for cargo visualization). Scale bars are 0.5 μm, and images were digitally magnified 100×. (C) DLS size distribution of cargo-loaded vesicles composed of mCherry-Z_E_ and a mixture of Z_R_-ELPs using a 0.3 Z_E_/Z_R_ ratio and 30 μM total ELP using a 12 Y_5_-Z_R_-ELP: 1 H_15_-Z_R_-ELP molar ratio with and without 10 μM of sfGFP(+10) cargo in 0.15 M NaCl or with HIP sfGFP(+10) OA or SD. (D) Epifluorescence images of structures composed of larger vesicles using a 0.05 Z_E_/Z_R_ ratio (all other conditions are the same as (A) for cargo visualization). Scale bars are 0.5 μm, and images were digitally magnified 100×.

HIP has been used to increase polymer- and lipid-based nanocarrier EE by increasing cargo hydrophobicity.^13^ Delivery of HIP loaded CytC by protein vesicles indicates that HIP is possibly changing the nature of cargo loading and release or the ability of cargo to be internalized by cells. In order to characterize how HIP enables vesicle loading and cytosolic delivery of biomacromolecules, two model superfolder green fluorescent protein (sfGFP) cargos were utilized. Negatively and positively surface-charged sfGFP model cargo proteins were expressed in *E. coli* and then purified (Figure S1).^34^ Cationic BA (Figure S2C, p*K*_a_ 9.36) was used to pair with sfGFP(−10), and no loss of protein fluorescence as a proxy for structure was observed (Figure S6D). The surface charge of sfGFP(−10) was influenced by complexation with BA, which resulted in an increase in zeta potential from −23.5 ± 2.6 to −15.7 ± 0.8 mV for 1:14 charge ratio sfGFP(−10) BA HIP complexes. The fact that excess ions were used makes it likely that not all ions could ion pair and that a significant fraction of them interact with proteins via hydrophobic interactions. Vesicles loaded with sfGFP(−10) without HIP or with 1 sfGFP(−10):14 BA charge ratio HIP (to match CytC) had similar size, PDI, and EE (Table 1 and Figure 3). Larger vesicles were made by decreasing the Z_E_/Z_R_ molar ratio to 0.05 in order to visualize the spatial distribution of the cargo in the vesicles, although there could be differences in smaller vesicles used for delivery due to tighter packing and smaller size with a 0.3 molar ratio. HIP loaded sfGFP(−10) vesicles have cargo loaded within the lumen distributed away from the membrane, while sfGFP(−10) vesicles without HIP have cargo-membrane colocalization (Figure 3B). We hypothesize that the difference in cargo distribution was due to the increase in cargo hydrophobicity and surface charge neutralization, which increased the hydrophobic interaction with the ELP and decreased the electrostatic attraction to excess Z_R_. Additionally, the zeta potential of sfGFP(−10) vesicles was −8.93 ± 0.77 mV and that of sfGFP(−10) BA HIP vesicles was −12.9 ± 1.0 mV. Despite the addition of cationic BA, sfGFP(−10) BA HIP vesicles have significantly more negative surface charge (*p* = 0.0055) than empty and sfGFP(−10) loaded vesicles. We hypothesize that this could be due to less surface-exposed positive Z_R_ from changes in vesicle packing and organization caused by the hydrophobic HIP complex.

The same vesicle fabrication and characterization were performed for sfGFP(+10) loaded vesicles using OA and SD. Without counterion, sfGFP(+10) had a zeta potential of −3.4 ± 0.9 mV and when complexed with OA at a charge ratio of 1:35 had a zeta potential of −57.4 ± 2.2 mV. Protein vesicles loaded with sfGFP(+10) without HIP and 1 sfGFP(+10): 35 OA charge ratio HIP were stable with diameters of 261.8 ± 4.71 and 174.1 ± 1.45 nm, respectively (Table 1). A higher charge ratio of OA was used for sfGFP(+) than for CytC to be consistent with cell viability experiments by using the same mass of counterion for both CytC and sfGFP(+10). These results indicate that the impact of HIP loading on vesicle size does depend on the properties of the cargo protein itself. HIP loading of sfGFP(+10) with OA did result in a loss of less than 30% of sfGFP(+10) fluorescence (Figure S6). Using this ratio of OA to load sfGFP(+10) into vesicles significantly improved the EE by transiently increasing the cargo hydrophobicity (Table 1). The location of the sfGFP(+10) cargo changed less than that for sfGFP(−10) cargo when using HIP, likely because there was already electrostatic repulsion between sfGFP(+10) and the positively charged excess Z_R_ (Figure 3D). SD HIP loading was not as ideal as with OA. Even with a lower charge ratio (1 sfGFP(+10): 5.4 SD), HIP sfGFP(+10) loaded vesicles with SD resulted in unstable aggregates (Table 1), though sfGFP(+10) fluorescence was not impacted (Figure S6C).

**Figure 4:**
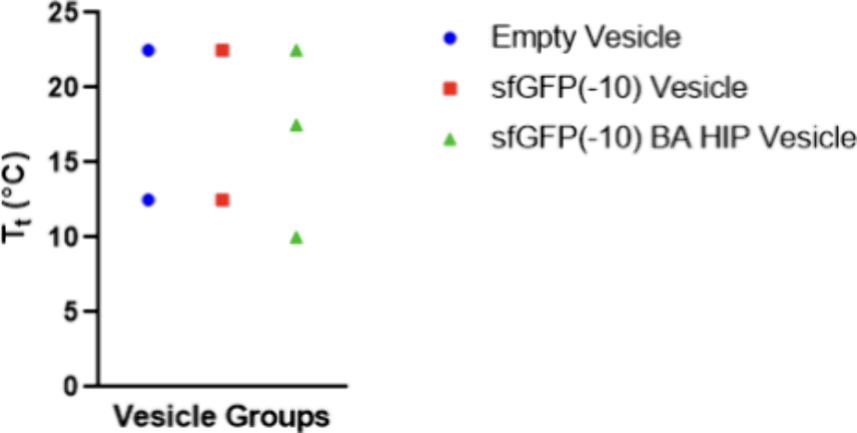
Effect of cargo and HIP on vesicle transition temperatures, *T*_t_. Transition temperatures of empty, sfGFP(−10), and sfGFP(−10) BA HIP vesicles with 10 μM sfGFP(−10) were determined from the midpoint of each linear region of the turbidity profile.

To perform more detailed structural characterization of HIP vesicles, sfGFP(−10) and sfGFP(−10) BA HIP vesicles were selected because of cargo fluorescence tracking ability, and BA is more easily dissolved than the anionic counterions. We first characterized the effect of cargo loading on the ELP properties. ELPs are thermoresponsive and exhibit lower critical solution temperature phase behavior when heated. This behavior is characterized by the transition temperature, *T*_t_, defined as the inflection point of the turbidity profile as a function of temperature. By measuring the *T*_t_ of empty, sfGFP(−10) loaded, and sfGFP(−10) BA HIP loaded vesicles, we found that vesicles undergo up to three transitions (Figures 4 and S8). Two are seen for all vesicles, related to the two different ELPs used in this work,^21,28^ and the third is only observed for HIP loaded vesicles. When multiple ELPs are present in a solution, there are multiple transition temperatures.^48^ Empty and sfGFP(−10) vesicles have the same *T*_t_ values, indicating that the sfGFP(−10) cargo does not impact *T*_t_. Wirtz et al. reported an increase in *T*_t_ when a pure hydrophobic ELP was diluted at a 1:1 molar ratio with a hydrophilic ELP.^49^ However, the vesicle formulation contains Z_R_-ELP (30 μM) and sfGFP(−10) (10 μM), which is likely insufficient dilution to alter *T*_t_. Conversely, the first *T*_t_ of sfGFP(−10) BA HIP vesicles is lower *T*_t_. BA is a hydrophobic salt that is likely responsible for the depression as both hydrophobicity and salt concentration influence the *T*_t_.^21,34^ HIP-cargo complexes used in this work include counterions above their respective critical micelle concentrations for pure counterion solutions. This could explain the observed third *T*_t_, although we did not detect micelles by DLS or SAXS.

To further analyze changes in vesicles with and without cargo and HIP, small-angle X-ray scattering (SAXS) was employed to characterize empty, sfGFP(−10) loaded, and sfGFP(−10) BA HIP loaded vesicles formed in PBS at 25 °C, then adjusted to pH 7.4 or pH 6, and incubated for 2 or 4 h before analyzing structures for 2 h per sample. TEM images of sfGFP(−10) BA vesicles at pH 7.4, 6.0, and 5.0 indicated that vesicles were still present at pH 6.0 after 2 h, but at pH 5.0, vesicles had merged and were no longer distinct structures (Figure S9). Thus, pH 6.0 was selected to study changes in the vesicle structure with acidic pH by SAX. Empty vesicles were best modeled using a combination of vesicle and cylinder models (Figure S9). The membrane thickness increased, and the cylinder (the shape of the protein amphiphiles) radius decreased at pH 6, suggesting that the ELP was relaxing or extending as histidine protonation disrupted hydrophobic interactions (Table 2). This agrees with our previous work using vesicles made from H_15_-Z_R_-ELP, where SAXS analysis revealed that the radius of gyration (*R*_g_) of a soluble protein increased at pH 5.5, indicating a more extended, hydrophilic soluble protein released from vesicle disassembly.^28^ However, vesicles loaded with sfGFP(−10) or sfGFP(−10) BA HIP scatter as fundamentally different objects than empty vesicles due to the contents in the lumen. They were fit with core–shell-sphere and monodisperse Debye Gaussian coil (MGC) models, indicating that the cargo changes the core scattering length density (Figure S9). sfGFP(−10) loaded vesicles demonstrated a decreased membrane thickness at pH 6, and sfGFP(−10) BA HIP vesicles exhibited no change in membrane thickness. Vesicles loaded with sfGFP(−10) have a significant increase in scaling for the MGC model, which represents a soluble protein, at pH 6 compared to pH 7.4 (Table 2). This suggests the release of cargo or vesicle proteins due to the start of vesicle disassembly. Conversely, sfGFP(−10) BA HIP loaded vesicles exhibited a trend of increased core–shell-sphere scaling at pH 6 at 4–6 h. Other samples (empty or sfGFP(−10) vesicles at all pHs or sfGFP(−10) HIP vesicles at pH 7.4) exhibited no change between 2 and 6 h. While the vesicles are too small to observe their disassembly by fluorescence microscopy,^28^ nanoparticle tracking analysis showed that at pH 6, sfGFP(−10) HIP loaded vesicles and sfGFP(−10) loaded vesicles both had an order of magnitude fewer particles indicating vesicle disassembly (Figure S10). Ristroph and co-workers loaded polymyxin B oleate HIP complexes into polymeric nanoparticles in water and found that at higher charge ratios (1:4), the nanoparticles have lamellar structures within the core that rearranged into an inverse hexagonal phase upon exposure to ions in PBS.^15^ At lower charge ratios (1:1), no change in assembly occurred. We hypothesize that a similar effect could occur, and the vesicle structure changes in part due to a change in HIP structure upon a change in pH given the high charge ratios used to load vesicles. We hypothesize that the increase in core–shell-sphere scaling could result from the release of HIP cargo from disassembling vesicles resulting in fewer vesicles but more core–shell spheres, which are below the nanoparticle tracking analysis size limit of 30 nm.

**Table 2:** SAXS Model Fitting Parameters for Empty, sfGFP(−10) Loaded, and sfGFP(−10) BA HIP Loaded Vesicles^a^

vesicle group	pH	*T* (h)	χ^2^	membrane thickness (Å)	cylinder radius (Å)	*R*_g_ (Å)	core SLD (10^–6^/Å^2^)	vesicle scale	cylinder scale	MGC scale	CSS scale
empty	7.4	2–6	1.75	202 ± 1.6	8.91 ± 0.29	n/a	n/a	2.07 ± 0.05	5.40 ± 0.34	n/a	n/a
	6.0	2–6	2.12	213 ± 1.3	7.30 ± 0.39	n/a	n/a	2.29 ± 0.04	6.12 ± 0.69	n/a	n/a
sfGFP(−10)	7.4	2–6	1.42	220 ± 5.6	n/a	24.1 ± 1.3	0.387 ± 0.01	n/a	n/a	0.219 ± 0.02	4.33 ± 0.58
	6.0	2–6	1.77	205 ± 3.6	n/a	24.6 ± 0.45	0.390 ± 0.01	n/a	n/a	0.272 ± 0.01	4.97 ± 0.27
sfGFP(−10) BA HIP	7.4	2–6	1.80	208 ± 9.4	n/a	21.1 ± 1.3	0.395 ± 0.01	n/a	n/a	0.256 ± 0.01	5.39 ± 0.64
	6.0	2–4	1.47	198 ± 3.9	n/a	22.1 ± 0.73	0.398 ± 0.01	n/a	n/a	0.247 ± 0.01	5.31 ± 0.34
	6.0	4–6	1.32	205 ± 9.1	n/a	23.6 ± 1.1	0.415 ± 0.01	n/a	n/a	0.239 ± 0.01	8.46 ± 0.82

a Empty vesicles were fit using vesicle and cylinder models, sfGFP(−10) and sfGFP(−10) BA HIP vesicles were fit using core–shell and monodisperse Debye Gaussian coil (MGC) models. *T* represents time in hours, χ^2^ represents chi squared—a measure of model fitting, *R*_g_ represents the radius of gyration, core SLD represents the core scattering length density, vesicle scale represents the scaling of that model, cylinder scale represents the scaling of that model, MGC scale represents the scaling of that model, and CSS scale represents the scaling of the core–shell-sphere model.

### 2.4 Uptake of sfGFP(−10) and sfGFP(+10) by Vesicles and Influence on Viability

**Figure 5:**
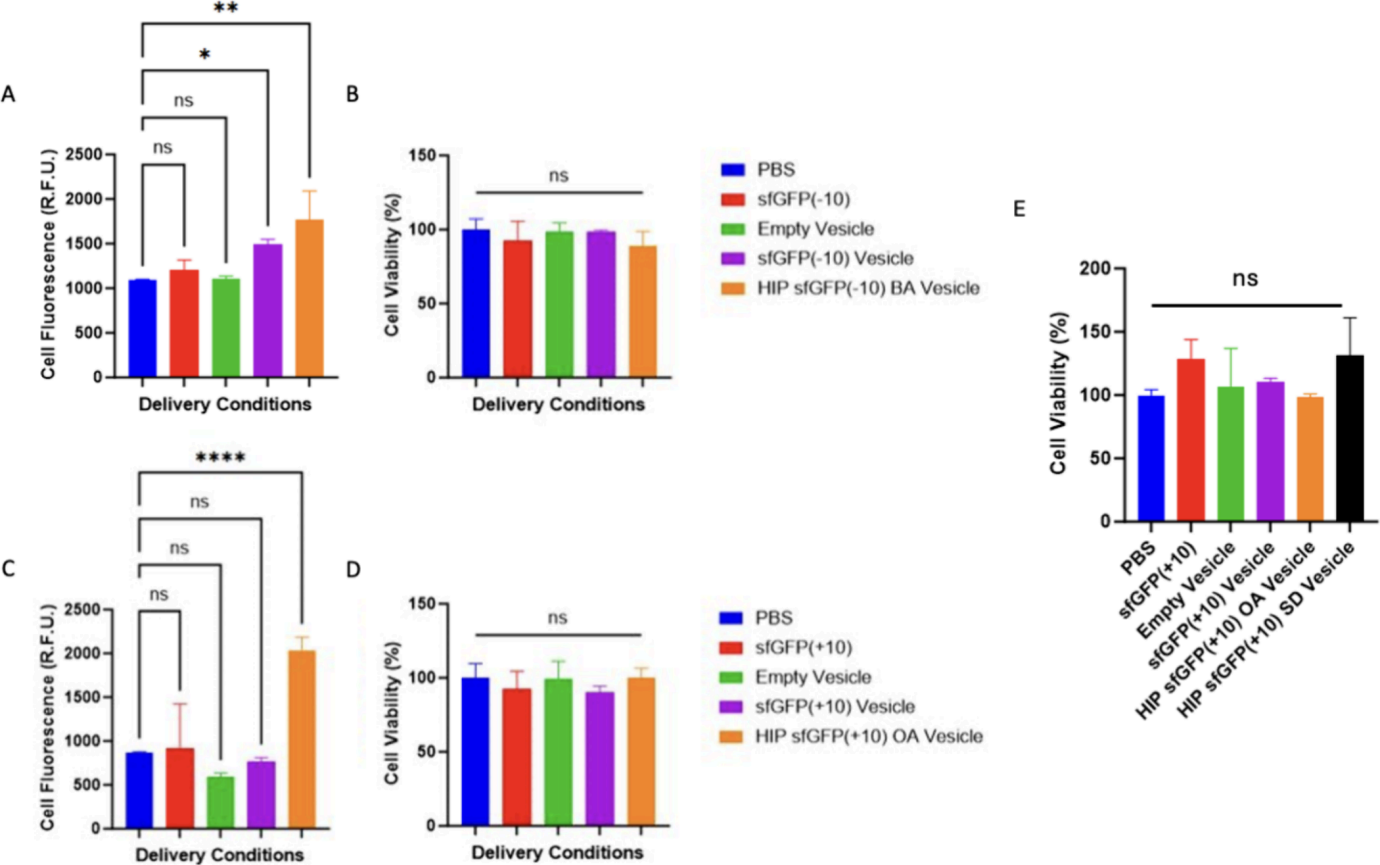
sfGFP(−10) and sfGFP(+10) loaded vesicle uptake and cellular viability. (A) Flow cytometry analysis showing median cell fluorescence of HeLa cells incubated with 1 μM sfGFP(−10) cargo for 24 h. (B) Viability 24 h after treatment using the MTT assay relative to media control. (C) Flow cytometry analysis showing median cell fluorescence of HeLa cells incubated with 1 μM sfGFP(+10) cargo for 24 h. (D) Viability 24 h after treatment using the MTT assay relative to media control. (E) Viability 48 h after treatment with 2 μM sfGFP(+10) loaded with 1 sfGFP(+10):16 OA and 1 sfGFP(+10):13 SD charge ratio using the MTT assay relative to media control. One-way ANOVA was utilized with *p* > 0.05 n.s., *p* < 0.05*, *p* < 0.01**, *p* < 0.0001****, and *n* = 3 groups with each experiment repeated at least twice. Error bars are standard deviation from the mean.

In order to measure the influence of HIP loaded cargos on viability, characterize uptake, and study membrane interactions, we used sfGFP(+10) and sfGFP(−10) to test anionic and cationic counterions, respectively. Vesicles with model cargos were delivered to HeLa cells for 24 h at a dose of 1 μM cargo, and trypan blue was used to quench extracellular fluorescence (Figure S6E). The mass of vesicles was adjusted to account for differences in EE. The following mass ratios were used: 1 sfGFP(+10) OA HIP: 0.653 sfGFP(+10): 0.207 sfGFP(−10) BA HIP: 0.168 sfGFP(−10). sfGFP(−10) vesicle and sfGFP(−10) BA HIP vesicle groups resulted in significant sfGFP(−10) uptake (Figure 5A). No protein vesicle loaded with sfGFP(−10) had any influence on HeLa cell viability, demonstrating that BA complexed with sfGFP(−10) in vesicles results in delivery without toxicity (Figure 5B). Using HIP to encapsulate sfGFP(+10) improved the EE significantly (Table 1), and sfGFP(+10) OA HIP loaded vesicles resulted in significantly higher HeLa cell uptake, while soluble sfGFP(+10) or vesicles loaded with sfGFP(+10) without HIP did not deliver any cargo (Figure 5C). Compared to sfGFP(−10) HIP vesicles, sfGFP(+10) HIP vesicles had a greater improvement in intracellular delivery, which could be due to the 2.5× higher charge ratio. However, CytC was delivered effectively with the same charge ratio used for sfGFP(−10), so there could also be effects from differences in the cargo charge and size. As with vesicles delivering sfGFP(−10), no protein vesicles carrying sfGFP(+10) had any influence on HeLa cell viability. OA complexed to sfGFP(+10) in vesicles improves delivery without toxicity (Figure 5D). It also serves as a control for CytC delivery experiments, demonstrating that CytC exerted the toxic effect shown in Figure 2, not OA.

**Figure 6:**
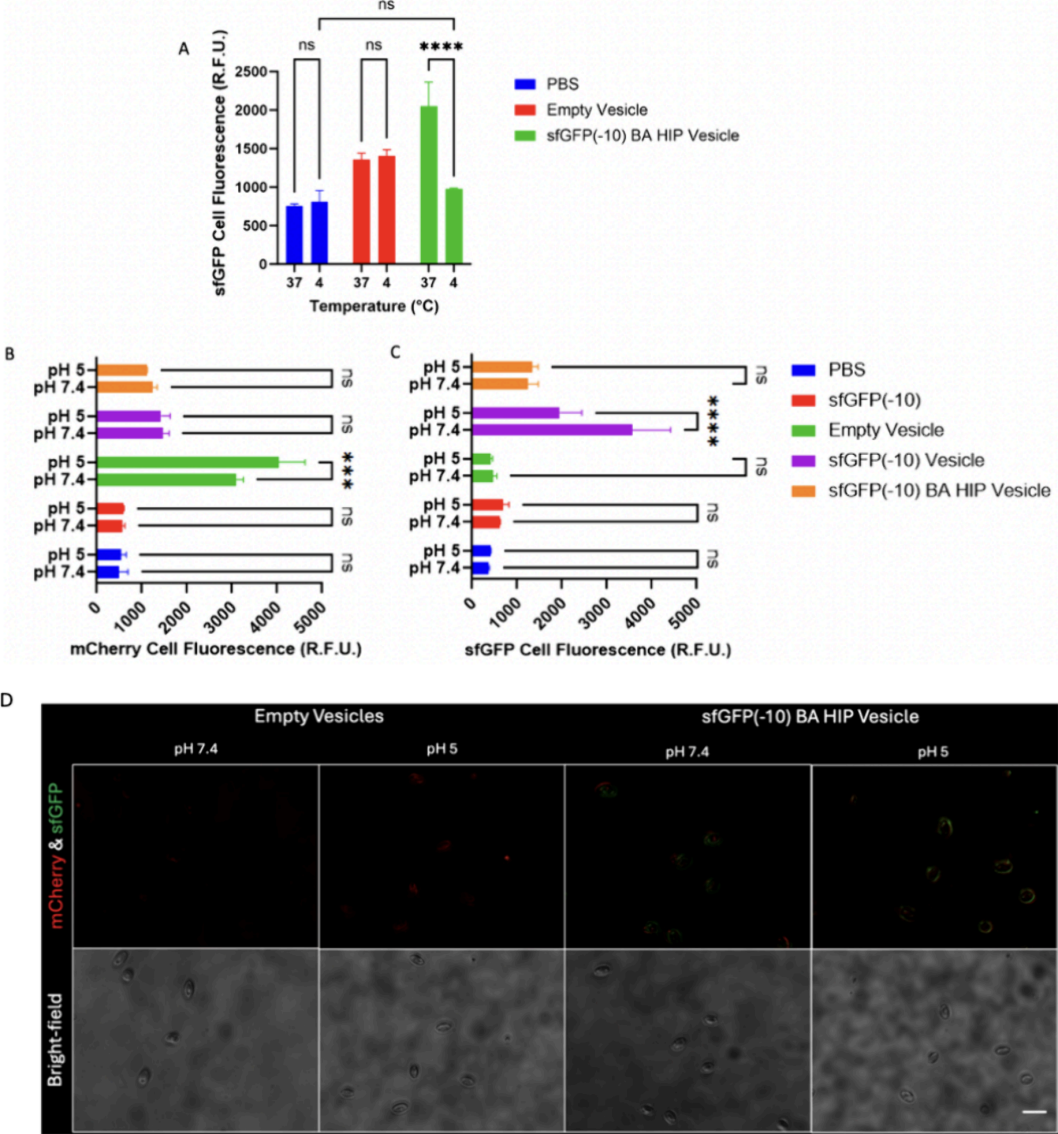
Energy-dependent endocytosis and RBC membrane interactions of sfGFP(−10) loaded vesicles. (A) Energy-dependent HeLa cell uptake using flow cytometry analysis of median fluorescence after 24 h of incubation with 1 μM sfGFP(−10). (B, C) RBC median fluorescence due to binding interactions with vesicles (B, mCherry fluorescence) or cargo (C, sfGFP fluorescence) at physiological and endosomal pH. 0.5% v/v RBCs were treated for 1 h, washed, and analyzed using flow cytometry and microscopy (scale bar: 20 μm). (D) Vesicles were composed of mCherry-Z_E_ and a mixture of Z_R_-ELPs using a 0.3 Z_E_/Z_R_ ratio and 30 μM total ELP with a 12 Y_5_-Z_R_-ELP: 1 H_15_-Z_R_-ELP molar ratio and 10 μM sfGFP(−10) cargo in 0.15 M NaCl and then treated according to the EE (using 0.413 or 0.51 mg/mL total vesicle proteins) to HeLa or RBC at 1 μM sfGFP. Two-way ANOVA was utilized with *p* > 0.05 n.s., *p* < 0.001***, *p* < 0.0001****, and *n* = 3 groups with each experiment repeated at least twice. Error bars are standard deviation from the mean.

To determine how vesicles deliver cargo into the cytosol, we measured uptake at 37 and 4 °C. Uptake was completely inhibited at 4 °C, indicating an energy-dependent uptake mechanism such as endocytosis (Figure 6A). We incubated vesicles with red blood cells (RBCs) to identify any vesicle membrane interactions, particularly at an acidic pH found in endosomes. RBCs are not endocytic; therefore, any cargo or vesicle fluorescence increase in RBCs indicates membrane binding or disruption. When pH-responsive vesicles disassemble at low pH, they fuse with one another before completely disassembling.^28^ It is possible that they could fuse with a membrane in a similar manner. Lipid nanoparticles merge with endosomal membranes in order to deliver cargos into the cytosol and exhibit improved cargo delivery when membrane fusion increases by use of ionizable lipids or cholesterol.^50,51^ Empty vesicles exhibited enhanced vesicle membrane-RBC interaction (mCherry fluorescence) at pH 5 compared to pH 7.4 (Figure 6B) suggesting that pH-induced destabilization of vesicles increased membrane interactions. Conversely, neither of the cargo-loaded vesicles had a pH-dependent change in RBC interaction, measured by mCherry fluorescence. It is possible that 1 h incubation was not sufficient to destabilize cargo-loaded vesicles, and SAXS data indicated that empty vesicles respond differently to acidification than loaded vesicles. However, RBCs exposed to sfGFP(−10) loaded vesicles without HIP exhibited sfGFP fluorescence at pH 7.4 that dropped significantly at acidic pH. This could indicate that while the vesicle-RBC interactions were pH insensitive for cargo-loaded vesicles, the interaction between the vesicle and cargo itself was pH sensitive without HIP and may have promoted the release of sfGFP(−10) from vesicles.

## 3 Conclusions

In this work, a mixed ELP protein vesicle formulation, where one ELP imparts increased stability and the other yields pH-responsiveness, was developed and characterized for biomacromolecule cargo delivery. HIP loaded vesicles enabled the improved uptake of both positively and negatively charged proteins without influencing cellular viability by using counterions with one charge group. We demonstrated that vesicles deliver CytC into the cytosol when using HIP above a critical charge ratio, resulting in a significant reduction in cancer cell viability. HIP loading of cargo was essential to successful delivery by protein vesicles and changed vesicle self-assembly, cargo location within the vesicle, and cargo-membrane interactions at acidic pH. The combination of protein vesicles and HIP loading broadened the types of cargo delivered using protein vesicles, and future work can explore the delivery of more cargo types both in vitro and in vivo.

## 4 Experimental Section/Methods

### 4.1 Expression and Purification of Fusion Proteins

The pET28a-sfGFP-His plasmids with charged sfGFP variants were purchased from Genscript. sfGFP variants were expressed in *E. coli* BL21 (DE3) star. To express these proteins, 1 L of lysogeny broth (LB) containing 50 mg of kanamycin was inoculated with 5 mL of overnight culture at 37 °C and then induced with 1 mM isopropyl ß-d-1 thiogalactopyranoside (IPTG) when the optical density at 600 nm was greater than 0.7. After 5 h of expression, cultures were collected by centrifugation at 4000 g for 10 min. The pellets were resuspended in lysis buffer containing 300 mM NaCl, 50 mM NaH_2_PO_4_, and 10 mM imidazole and lysed by sonication. Next, the supernatant was collected after centrifugation at 10,000 relative centrifugal force (rcf) for 10 min and incubated with Ni-nitrilotriacetic acid (NTA) agarose resin (Qiagen) for at least 1 h at 4 °C. This mixture was loaded in an Econo-Column (Biorad), washed with 100 mL of 25 mM imidazole, and 10 mL of elution was collected using 250 mM imidazole. Purity was verified using sodium dodecyl sulfate polyacrylamide gel electrophoresis (SDS-PAGE), and then the proteins were buffer exchanged into PBS by dialysis using a 10 kDa MWCO membrane with three buffer exchanges at 4 °C.

The genes for H_15_-Z_R_-ELP and Y_5_-Z_R_-ELP were purchased from Genscript. mCherry-Z_E_ was created previously.^52^ These three proteins were expressed in AF-IQ BL21 (DE3) *E. coli* in 1 L of LB containing 200 mg of ampicillin and 34 mg of chloramphenicol and collected using centrifugation at 4000 rcf for 10 min. mCherry-ZE was purified using the same method as sfGFP. Z_R_-ELPs were purified by affinity chromatography with the same resin and columns as described above. Cells were lysed under denaturing conditions in 8 M urea, 100 mM NaH_2_PO_4_, and 10 mM TrisCl at pH 8, washed with 8 M urea at pH 6.3, and eluted into 6 M guanidine hydrochloride, 100 mM NaH_2_PO_4_, and 10 mM TrisCl, as in our past work.^20,27,28,34^ Y_5_-Z_R_-ELP purification used 5% n-octyl-ß-d-glucopyranoside in the lysis buffer as in our past work.^21^ After purification, the protein was buffer exchanged into Milli-Q water and freeze-dried for long-term use.

### 4.2 HIP Complex and Vesicle Formation

HIP complexes were formed by a simple mixing method. Cargo proteins at a concentration of 1 mg/mL in PBS (pH 7.4) were mixed with varying concentrations of counterion (in pure form, not salts) at equal volumes on ice before being added to vesicle proteins; the conditions used for each complex are listed in Table 1. CytC, sfGFP(−10), and sfGFP(+10) have surface charges of 8.085, −9.424, and 11.408, respectively, as found using two online tools.^38,53^ The formula for calculating the charge ratio is as follows:
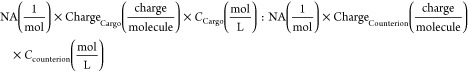


Vesicle solutions were mixed on ice starting with Milli-Q water, 30 μM Z_R_-ELPs, mCherry-Z_E_, cargo, and then 10× PBS to achieve a final salt concentration of 0.15 M. Unless otherwise stated, a Z_E_/Z_R_ ratio of 0.3 was utilized. The amount of cargo added was optimized based on the EE so that the maximum loading was achieved (depending on the cargo concentration between 6 and 10 μM).

### 4.3 Characterization Techniques for Vesicles

Turbidity was determined by measuring absorbance at 400 nm with a Biotek Instrument Synergy H4 Hybrid multimode microplate reader set to 25 °C as the ice-cold solution warmed for over an hour. The *T*_t_ was measured at an optical density of 400 nm every minute as the temperature increased from 5 to 40 °C using an Applied Photophysics Chirascan Plus CD UV–vis spectrophotometer. The *T*_t_ is the inflection point or points for a protein variant mixture determined by a region with a significant change in slope. Size and zeta potential were measured by DLS using Malvern Instruments Zetasizer NanoZS with a 4 mW He–Ne laser at 633 nm wavelength to detect backscattering (173°) using PBS at pH 7.4 solvent conditions and protein material selection for DLS or 0.1× PBS at pH 7.4 solvent conditions and protein material selection for zeta potential. Average values were used for vesicle size. Electrophoretic mobility was converted to zeta potential using the Smoluchowski approximation. The EE of protein vesicles was determined using 100 kDa MWCO 1 mL centrifugal filters to separate vesicles from unencapsulated cargo by loading vesicles in PBS and then centrifuging at 12,500 rcf for 10 min. Fluorescence and absorbance calibration curves were evaluated for each sfGFP charge variant and CytC using the same microplate reader used for turbidity measurements. Filtrate and retentate fluorescence or absorbance was measured to calculate EE (retentate divided by the sum of retentate and filtrate) where the manufacturer reports fewer than 10% of protein remains when smaller than the MWCO.^54^

Fluorescence imaging of vesicles was performed using an epifluorescence microscope (Zeiss Axio Observer Z1) using a 100× oil immersion lens. For high-magnification imaging, transmission electron microscopy (TEM), using a JEOL 100CX-II and imaged at 100 kV, grids were prepared using 10 μL sample on copper grids, letting the sample adhere for 5 min, washing in water for 30 s, staining with 1% phosphotungstic acid for 10 s, washing again for 30 s, and drying overnight. SAXS data were collected using a Rigaku BioSAXS 2000 instrument operated by the Center for Structural Molecular Biology at the Oak Ridge National Laboratory. The wavelength was 1.542 Å using momentum transfers 0.008 < *Q* < 0.70 Å^–1^. SAXSLab 4.0.2 software corrected for solvent background scattering, and then data analysis was performed using SasView software (https://sasview.org). Data between 0.008 and 0.35 Å^–1^ were fit. Nanoparticle tracking analysis data were collected using a Nanosight NS300.

### 4.4 Cell Culture and Cargo Uptake

HeLa cells (ATCC; taken from Henrietta Lacks, http://henriettalacksfoundation.org/) were seeded at 15,000 cells/well using Dulbecco’s modified Eagle’s medium (DMEM) supplemented with 10% fetal bovine serum (FBS) in 96-well plates for 24 h prior to treatment, washed with PBS, then treated with sfGFP- or CytC-loaded vesicles and controls in media containing DMEM and 10% FBS at 37 °C in a humidified environment containing 5% CO_2_. K-562 (human erythromyeloblastoid leukemia cell line ATCC, UK; CCL-243), originally derived from a patient with the blast crisis (acute leukemia phase) of chronic myeloid leukemia, was chosen to model acute myeloid leukemia. For 2D experiments, cells were seeded at 25,000 cells/well in 96-well plates for 24 h prior to treatment in Roswell Park Memorial Institute 1640 Medium (RPMI; Corning, Manassas, VA) supplemented with 10% v/v FBS (heat-inactivated; Gibco, NY), washed with PBS, then treated with vesicles in supplemented media at 37 °C in a humidified environment containing 5% CO_2_ for 48 h. For 3D experiments, 100 μL of cell suspension (4 × 10^6^ cells/scaffold) was seeded onto the scaffolds, placed in 24-well tissue culture plates, and incubated for 30 min at 37 °C in an atmosphere of 5% CO_2_ to allow cell adhesion prior to adding 1.5 mL of culture medium. Scaffolds were cultured for 7 days with full media exchange every other day prior to adding the vesicles. NIH 3T3 fibroblast cells expressing eGFP (Cell BioLabs) were seeded at 35,000 cells/well in a 96-well plate for 24 h before treatment using DMEM supplemented with 10% FBS. All groups were treated in the presence of serum-containing media and cultured at 37 °C in an atmosphere of 5% CO_2_.

### 4.5 Scaffold Preparation

Scaffolds were fabricated by thermally induced phase separation as previously described.^46,55,56^ Briefly, polyurethane pellets (Estane 58300; Velox, Germany) were dissolved in dioxane (99.8% pure, Merck Millipore, Germany) to form a polymer solution (5 wt %) that was frozen at −80 C with subsequent sublimation in an ethylene glycol bath at −15 °C. The scaffolds (pore size 100–250 um, porosity 90–95%) were cut into 5 × 5 × 5 mm cubes and prewetted in ethanol (70% v/v, Thermo Scientific) for 1 min, then washed in PBS for 20 min, and centrifuged for 10 min at 2500 rpm in PBS. Scaffolds were coated with collagen type I from calf skin (Sigma-Aldrich). Cubes were transferred into a 62.5 μg/mL collagen solution solubilized in 0.1 M acetic acid and dissolved in deionized water at pH 7 (readjusted with addition of 0.1 M NaOH) and centrifuged at 2000 rpm for 20 min in the protein solution followed by a final centrifugation step in PBS at 1500 rpm for 10 min to unblock the surface pores. Scaffolds were sterilized by UV sterilization (8 min exposure at 230 V, 50 Hz, 0.14 A, UV lamp, Thermo Fisher) and 2 h immersion in ethanol (70%). The collagen-coated scaffolds were washed twice for 15 min in PBS before adding media and placed in a humidified incubator for 3 days at 37 °C and 5% CO_2_ prior to seeding with leukemia cells.

### 4.6 Cargo Internalization

Following incubation with sfGFP-loaded vesicles and controls, cells were detached by using trypsin and resuspended in PBS with trypan blue to quench extracellular fluorescence. The green fluorescence of 10,000 cells was measured using 488 nm excitation on the CytoFLEX flow cytometer (BD Biosciences). Gating was done on PBS groups to isolate single cells and compare the increase or decrease in GFP signal (Figure S11).

### 4.7 Cell Viability

The in vitro toxicity of vesicles containing the different cargos was quantified using an MTT assay. After vesicle incubation, the media was removed, cells were washed with PBS, and then fresh media with 10 μL of 5 mg/mL MTT solution (Biotium) was added. The cells were incubated for 4 h at 37 °C in a humidified environment containing 5% CO_2_. Then, 200 μL of dimethyl sulfoxide (DMSO) was added to dissolve the formazan crystals. Absorbance of solutions was measured at 570 and 630 nm. Cell viability was calculated by using an absorbance ratio in a treated group relative to the PBS control group. Similarly, an MTS assay was performed on scaffolds, which is analogous to an MTT assay minus the solubilization step.

### 4.8 Endosomal Membrane Interactions

Washed 5% turkey RBCs from Fisher Scientific were diluted to a 0.5% v/v concentration in PBS. 100 μL of diluted nanoparticles were incubated with 10 μL of RBCs at 37 °C for 1 h in a 96-well plate in a humidified environment containing 5% CO_2_. For membrane binding experiments, 100 μL of RBCs were plated and then treated with nanoparticles for 1 h at 37 °C in a 96-well plate in a humidified environment containing 5% CO_2_. After incubation with treatment groups, the RBCs were washed with PBS and then analyzed using flow cytometry to measure the mCherry or sfGFP signal as described above.

## Supporting Information Available

Z_R_-ELP protein sequences, SDS-PAGE gels of purified proteins, hydrophobic ion structures and LogP values, vesicle diameter and turbidity data, cell viability data from different CytC vesicles, effect of HIP on protein fluorescence, transition temperature profiles, SAX model fitting, TEM and nanoparticle tracking analysis of vesicles at different pH values, and example gating strategy for flow cytometry data (PDF)
