# Supplementary material for: Intracellular Biomacromolecule Delivery by Stimuli-Responsive Protein Vesicles Loaded by Hydrophobic Ion Pairing
Source: ACS Omega. 2025 Jan 14;10(3):2628–39. doi: 10.1021/acsomega.4c07666 (PMC11780410; doi:10.1021/acsomega.4c07666)
Supplement: Supplementary file 1 — ao4c07666_si_001.pdf [file ao4c07666_si_001.pdf]

Supporting Information

Intracellular biomacromolecule delivery by stimuli responsive protein vesicles loaded by hydrophobic ion pairing

Mikaela A. Gray[A]*, Alejandro de Janon*[B]*, Michelle Seeler*[C]*, William T. Heller*[D]*, Nicki Panoskaltsis*[B,E,F]*, Athanasios Mantalaris*[B,E,G]*, and Julie A. Champion*[A]*

A. Chemical and Biomolecular Engineering Georgia Institute of Technology Atlanta GA, USA.
E-mail: Julie.champion@chbe.gatech.edu

B. Biomedical Systems Engineering Laboratory Georgia Institute of Technology Atlanta GA, USA.

C. School of Biological Sciences Georgia Institute of Technology Atlanta GA, USA.

D. Neutron Scattering Division, Oak Ridge National Laboratory Oak Ridge, TN, USA.

E. School of Pharmacy & Pharmaceutical Sciences, Trinity College Dublin, Dublin, Ireland.

F. Department of Haematology, St. James's Hospital, Dublin, Ireland.

G. National Institute for Bioprocessing Research and Training, Dublin, Ireland.

Table S1. ((Sequences of ELP variants. Subscripts in the ELP sequence column indicate number of repeats. Bolded amino acids are modifications to the original ELP sequence, in place of valines.))

ELP NAME	ELP SEQUENCE
**Y_5-Z_R-ELP**	[VPGVG VPG**Y**G VPGFG VPGVG VPGVG]$_5$
**H_{15}-Z_R-ELP**	[VPG**H**G VPG**H**G VPGFG VPG**H**G VPGVG]$_5$

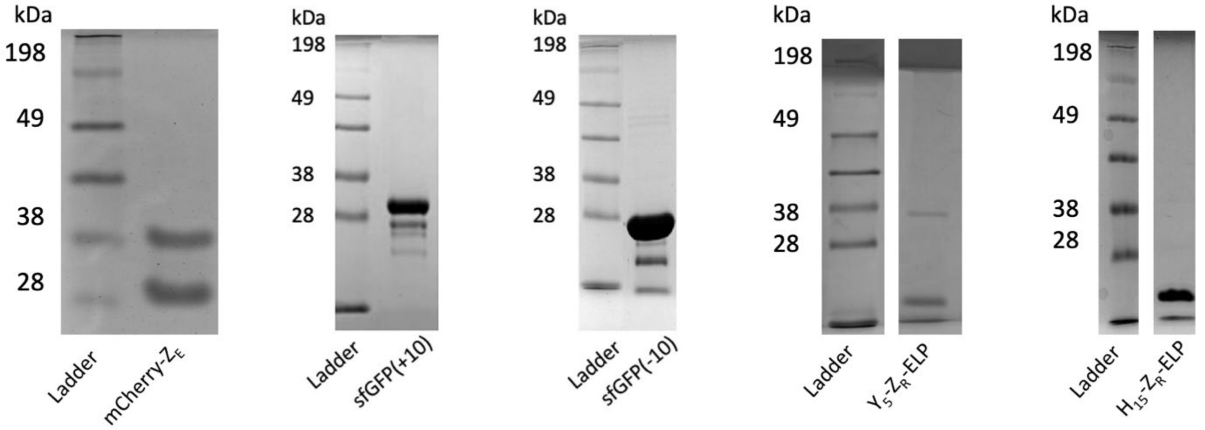

Figure S1. SDS-page gels of mCherry-Z_E, sfGFP(-10), sfGFP(+10), Y_5-Z_R-ELP, and H_{15}-Z_R-ELP proteins. mCherry-ZE and the sfGFP samples when boiled results in multiple populations one for the folded fluorescent protein and the other for the partially folded protein.[46] The boiling hydrolyzes the N=C bond resulting in the decrease in fluorescence.[49] Y_5-Z_R-ELP has two bands where the lower band is protein monomer and the higher molecular weight band is the dimer. Dimers form due to the disulfide bonds between the terminal cysteine residues in each protein. The lowest band in Y_5-Z_R-ELP and H_{15}-Z_R-ELP gels is the dye front.

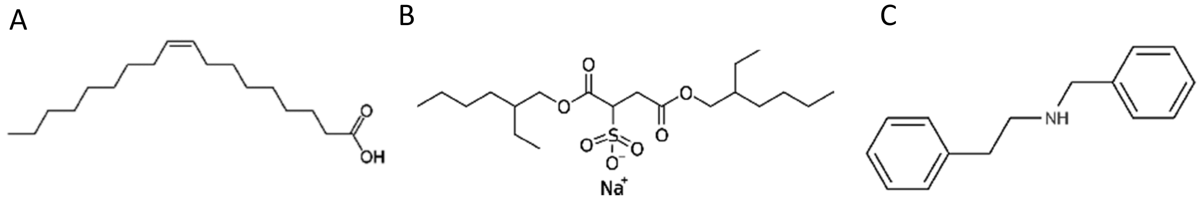

Figure S2. Structures of counterion (A) OA with a LogP=6.78, (B) SD with a LogP=5.2, and (C) BA with a LogP=3.6.[11]

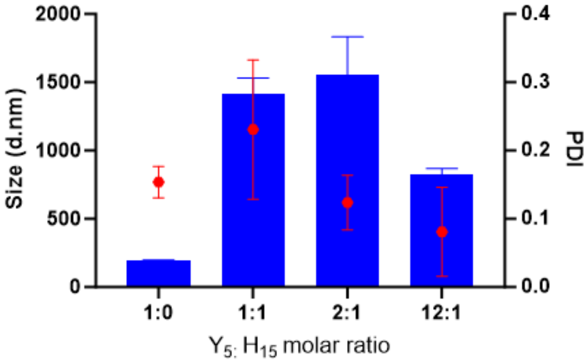

Figure S3. Protein vesicle hydrodynamic diameter and polydispersity index (PDI) of varying Y_5-Z_R-ELP: H_{15}-Z_R-ELP molar ratios. Blue bars represent size and red dots are PDI. Vesicles were composed of 0.15 M NaCl, 0.05 Z_E/Z_R ratio, and 30 μM total ELP.

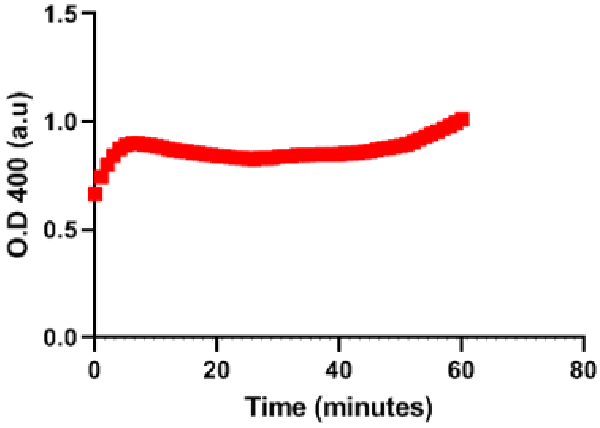

Figure S4. Turbidity profile of 0.15 M NaCl, 0.3 Z_E/Z_R ratio, and 30 μM total ELP using a 12 Y_5-Z_R-ELP: 1 H_{15}-Z_R-ELP molar ratio solution over 1 hour after removing from ice to room temperature.

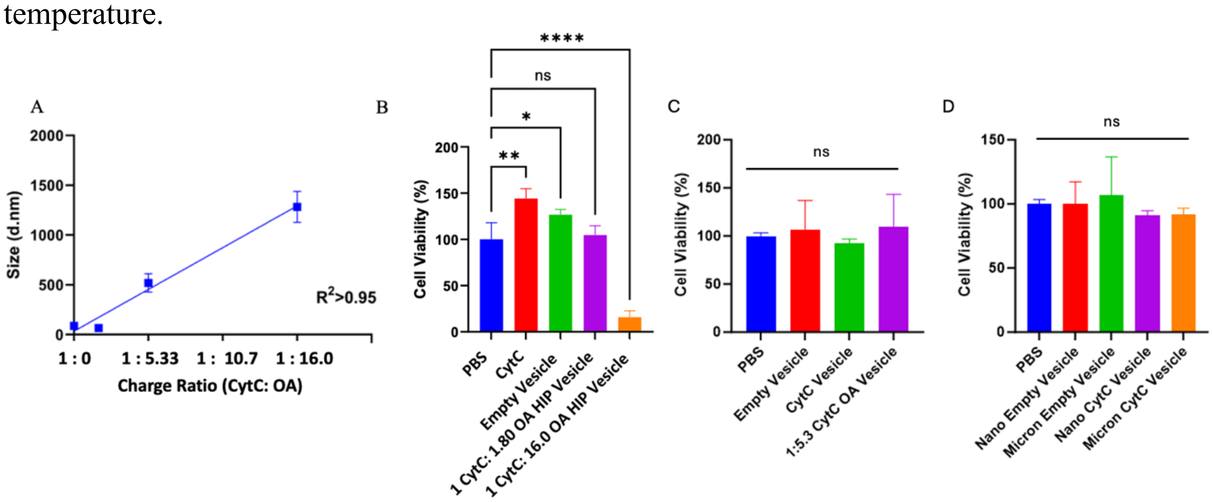

Figure S5. Characterization of HIP CytC OA vesicles with varying amounts of OA. (A) Hydrodynamic diameter of 0.15 M NaCl, 0.3 Z_E/Z_R ratio, and 30 μM total ELP using a 12 Y_5-Z_R-ELP: 1 H_{15}-Z_R-ELP molar ratio solution protein vesicles loaded with the same concentration of CytC with varying concentrations of OA, (B) viability of 2D K562 cells treated with CytC vesicle groups including two charge ratios of HIP, (C) viability of HeLa cells treated with CytC vesicle and HIP vesicle groups, and (D) viability of HeLa cells treated with CytC loaded nano (0.3 Z_E/Z_R ratio) and micron (0.05 Z_E/Z_R ratio) sized vesicles without any counterion. Groups used 2 μM CytC and 48 hour treatment. One-way ANOVA was

utilized with p>0.05 n.s, p<0.05*, p<0.01**, p<0.0001****, and n=3 groups with each experiment repeated at least twice. Error bars are standard deviation from the mean.

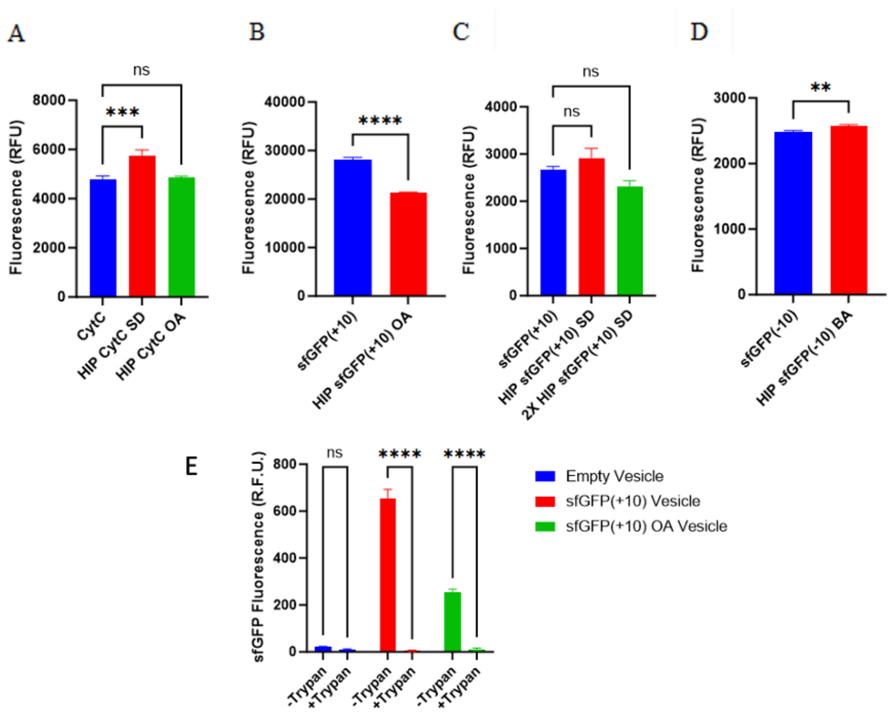

Figure S6. Counterion influence on (A) CytC, (B) sfGFP(+10) & (C) sfGFP(+10) and (D) sfGFP(-10) cargo fluorescence as a measure of change in protein structure. Trypan vesicle sfGFP quenching using 2 µM sfGFP(+10) vesicles and 2 µM sfGFP(+10) loaded by 1 sfGFP(+10) :16 OA charge ratio vesicles (exact conditions used for uptake experiments) diluted with 100 µL PBS(-Trypan) or trypan blue (+Trypan) (E). Fluorescence measured with a plate reader. One-way ANOVA was utilized in (A), (C), and (E) and a t-test was utilized in (B) and (D) with p>0.05 n.s, p<0.01**, p<0.001***, p<0.0001****, and n=3 groups with each experiment repeated at least twice. Error bars are standard deviation from the mean.

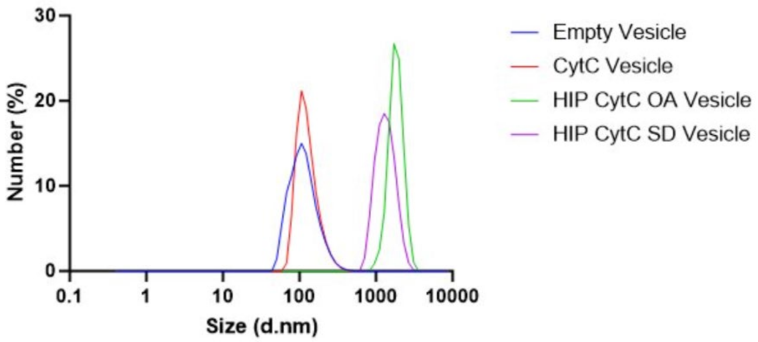

Figure S7. DLS of empty, CytC, and HIP CytC vesicles by number %. Vesicle solutions consisted of mCherry-Z_E and a mixture of Z_R-ELPs using a 0.3 Z_E/Z_R ratio, 30 µM total ELP with a 12 Y_5-Z_R-ELP: 1 H_{15}-Z_R-ELP molar ratio, 10 µM CytC cargo, and 0.15 M NaCl.

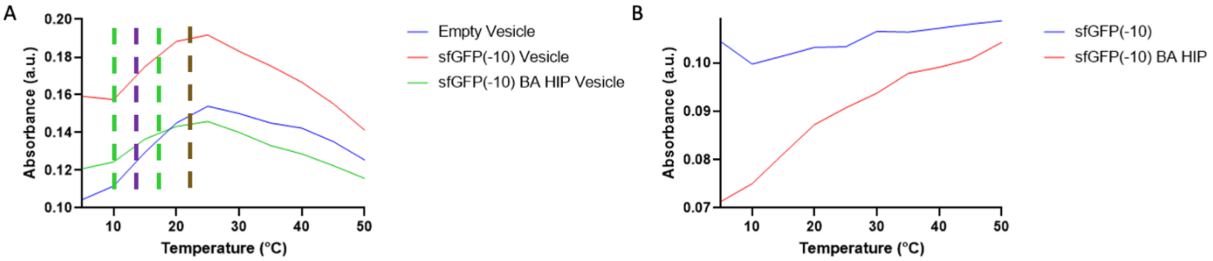

Figure S8. Transition temperature profiles of A) empty, sfGFP(-10), and sfGFP(-10) BA HIP vesicles with 10 μM sfGFP(-10) with a 1 degree/minute ramp rate. Green dotted lines locate T_t values of sfGFP(-10) BA HIP vesicle, purple dotted line identifies sfGFP(-10) vesicle and empty vesicle T_t values, and brown dotted line lies on the T_t for all three groups. Transition temperature profiles of B) sfGFP(-10) and sfGFP(-10) BA HIP solutions with 10 μM sfGFP(-10) with a 1 degree/minute ramp rate.

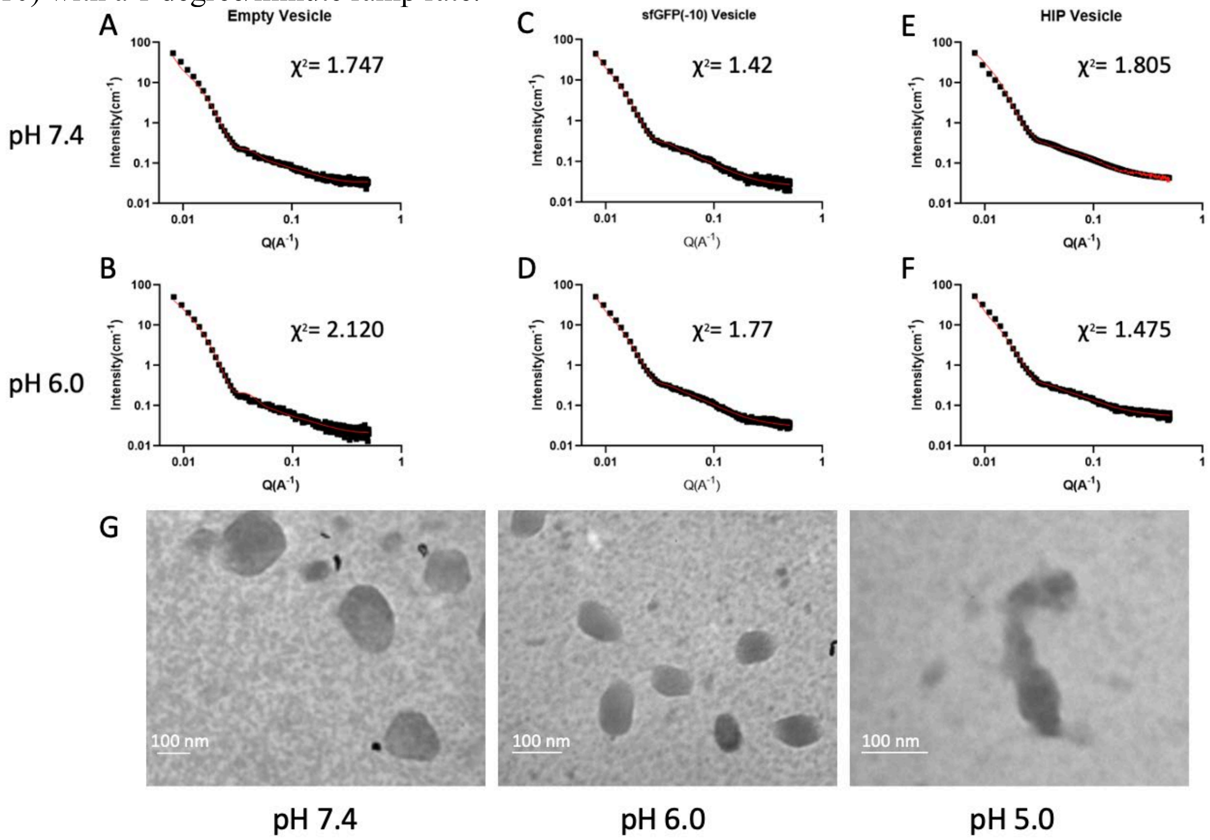

Figure S9. SAXS model fitting of empty protein vesicles (A) at pH 7.4 and (B) pH 6.0, (C) sfGFP(-10) vesicles at pH 7.4 and (D) pH 6.0, and (E) sfGFP(-10) BA HIP vesicles at pH 7.4 and (F) pH 6.0. TEM images of HIP sfGFP(-10) BA loaded vesicles diluted at varying pH values for 2 hours prior to staining and drying on sample grid (G). Loaded groups each had 10 μM sfGFP(-10) and 0.816 mg/mL protein vesicle.

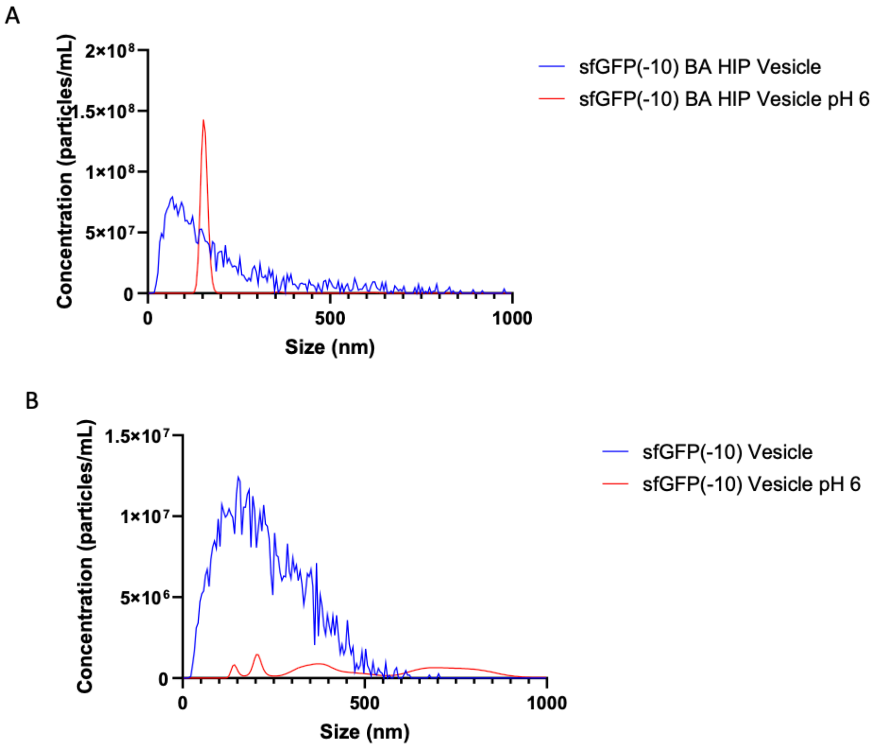

Figure S10. NTA analysis of loaded vesicles. (A) sfGFP(-10) and (B) sfGFP(-10) BA vesicles at pH 7.4 and pH 6. Vesicles were diluted by a factor of 10 to a total protein concentration of 0.0816 mg/mL.

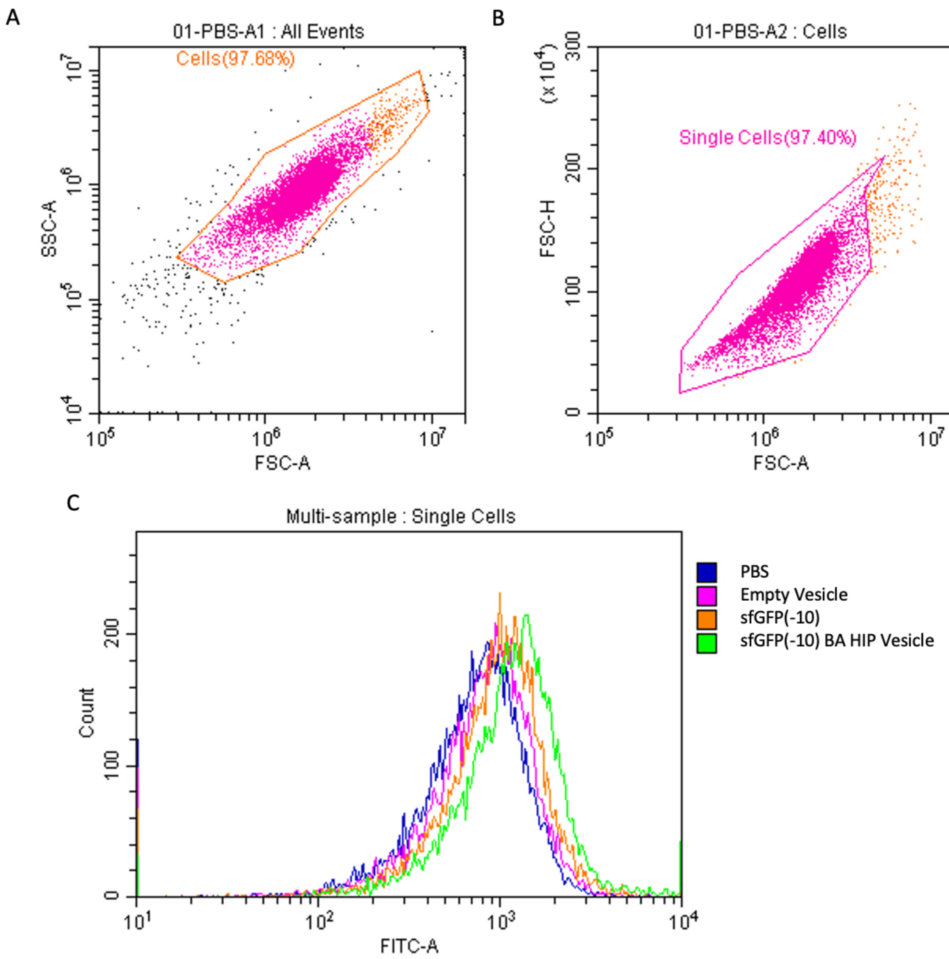

Figure S11. Example flow cytometry gating to select single cell populations. (A) Forward scatter area (FSC-A) and side scatter area (SSC-A) enable selection of cell populations. (B FSC-A and FSC height (FSC-H) enable selection of single cell populations by drawing tight gates to remove doublets, gated using a linear scale. (C) Single cell populations were used to measure sfGFP uptake using median values.
